# The presymptomatic and early manifestations of semantic dementia

**DOI:** 10.1093/brain/awaf351

**Published:** 2025-09-23

**Authors:** David J Whiteside, Matthew A Rouse, P Simon Jones, Ian Coyle-Gilchrist, Alexander G Murley, Katherine Stockton, Laura E Hughes, Richard A I Bethlehem, Varun Warrier, Matthew A Lambon Ralph, Timothy Rittman, James B Rowe

**Affiliations:** Department of Clinical Neurosciences, University of Cambridge, Cambridge CB2 0SZ, UK; Department of Neurology, Cambridge University Hospitals NHS Foundation Trust, Cambridge CB2 0QQ, UK; Medical Research Council Cognition and Brain Sciences Unit, University of Cambridge, Cambridge CB2 7EF, UK; Department of Clinical Neurosciences, University of Cambridge, Cambridge CB2 0SZ, UK; Department of Neurology, Norfolk and Norwich University Hospital NHS Trust, Norwich NR4 7UY, UK; Department of Clinical Neurosciences, University of Cambridge, Cambridge CB2 0SZ, UK; Department of Neurology, Cambridge University Hospitals NHS Foundation Trust, Cambridge CB2 0QQ, UK; Department of Clinical Neurosciences, University of Cambridge, Cambridge CB2 0SZ, UK; Department of Neurology, Cambridge University Hospitals NHS Foundation Trust, Cambridge CB2 0QQ, UK; Medical Research Council Cognition and Brain Sciences Unit, University of Cambridge, Cambridge CB2 7EF, UK; Department of Psychology, University of Cambridge, Cambridge CB2 3EB, UK; Department of Psychology, University of Cambridge, Cambridge CB2 3EB, UK; Department of Psychiatry, University of Cambridge, Cambridge CB2 0SZ, UK; Medical Research Council Cognition and Brain Sciences Unit, University of Cambridge, Cambridge CB2 7EF, UK; Department of Clinical Neurosciences, University of Cambridge, Cambridge CB2 0SZ, UK; Department of Neurology, Cambridge University Hospitals NHS Foundation Trust, Cambridge CB2 0QQ, UK; Department of Clinical Neurosciences, University of Cambridge, Cambridge CB2 0SZ, UK; Department of Neurology, Cambridge University Hospitals NHS Foundation Trust, Cambridge CB2 0QQ, UK; Medical Research Council Cognition and Brain Sciences Unit, University of Cambridge, Cambridge CB2 7EF, UK

**Keywords:** semantic dementia, semantic behavioural variant frontotemporal dementia, semantic variant primary progressive aphasia, machine learning classification

## Abstract

People with semantic dementia (SD) or semantic variant primary progressive aphasia typically present with marked atrophy of the anterior temporal lobe, and thereafter progress more slowly than other forms of frontotemporal dementia. This suggests a prolonged prodromal phase with accumulation of neuropathology and minimal symptoms, about which little is known. To study early and presymptomatic SD, we first examine a well-characterized cohort of people with SD recruited from the Cambridge Centre for Frontotemporal Dementia. Five people with early SD had coincidental MRI prior to the onset of symptoms or were healthy volunteers in research with anterior temporal lobe atrophy as an incidental finding. We model longitudinal imaging changes in left- and right-lateralized SD to predict atrophy at symptom onset. We then assess 61 203 participants with structural brain MRI in the UK Biobank to find individuals with imaging changes in keeping with SD but with no neurodegenerative diagnosis. To identify these individuals in the UK Biobank, we design an ensemble-based classifier, differentiating baseline structural MRI in SD from healthy controls and patients with other neurodegenerative diseases, including other causes of frontotemporal lobar degeneration. We train the classifier on a Cambridge-based cohort (SD *n* = 47, other neurodegenerative diseases *n* = 498, healthy controls *n* = 88) and test it on a combined cohort from the Neuroimaging in Frontotemporal Dementia study and the Alzheimer’s Disease Neuroimaging Initiative (SD *n* = 42, other neurodegenerative disease *n* = 449, healthy controls *n* = 127).

From our case series, we find people with marked atrophy 3 to 5 years before recognition of symptom onset in left- or right-predominant SD. We present right-lateralized cases with subtle multimodal semantic impairment, found concurrently with only mild behavioural disturbance. We show that imaging measures can reliably and accurately differentiate clinical SD from other neurodegenerative diseases (recall: 0.88, precision: 0.95, F1 score: 0.91). We find individuals with no neurodegenerative diagnosis in the UK Biobank with striking left-lateralized (prevalence ages 45–85, 4.8/100 000) or right-lateralized (5.9/100 000) anterior temporal lobe atrophy, with deficits on cognitive testing suggestive of semantic impairment. These individuals show progressive involvement of other cognitive domains in longitudinal follow-up. Together, our findings suggest that (i) there is a burden of incipient early anterior temporal lobe atrophy in older populations, with comparable prevalence of left- and right-sided cases from this prospective unbiased approach to identification; (ii) substantial atrophy is required for manifest symptoms, particularly in right-lateralized cases; and (iii) semantic deficits across multiple domains can be detected in the early symptomatic phase.

## Teaser

Whiteside *et al.* find that brain imaging can reveal temporal lobe atrophy related to semantic dementia around 3–5 years before symptom onset. Whereas clinical cohort studies have shown more people with left-predominant disease, this prospective analysis of a large population sample revealed an even split between left- and right-sided atrophy.

## Introduction

Semantic dementia (SD) is characterized by the progressive degradation of semantic knowledge for all types of concepts, across all modalities.^1-3^ Structural neuroimaging reveals atrophy of the anterior temporal lobes (ATLs),^4,5^ which is marked at first clinical presentation,^6,7^ even in the earliest cases in larger cohorts and case series.^6,8,9^ Disease progression is thereafter less rapid than for other types of frontotemporal dementia.^10^ This raises the likelihood of a long prodrome with minimal symptoms, despite the neuropathological burden, typically Tar DNA binding protein 43 (TDP-43) type C proteinopathy.^11-13^ Studying the presymptomatic or prodromal phase is challenging in a sporadic disease like SD, with no established genetic cause.^14-16^

ATL atrophy in SD is commonly lateralized at presentation. Those with left-predominant atrophy most often present with anomia and comprehension difficulties,^17,18^ meeting the diagnostic criteria for semantic variant primary progressive aphasia (svPPA).^19^ Although seemingly rarer to present to clinic in the early phase, case reports of patients with right-predominant atrophy highlight early deficits in familiar face recognition, followed by the emergence of a multimodal semantic impairment and behavioural changes.^6^ Diagnostic criteria for this right-lateralized homologue have recently been advanced, with the syndrome referred to variously as right semantic dementia, semantic behavioural variant frontotemporal dementia (sbvFTD)^20^ or right temporal variant of frontotemporal dementia (rtvFTD).^21^ An international working group has been formed, with the aim to define a cohesive clinical phenotype for this syndrome.^22^ In clinical case series^6,23,24^ and in studies of proven TDP-43 type C pathology^25^ there is a majority of patients with left-predominant atrophy at presentation. It is not known whether this apparent asymmetry reflects clinical ascertainment bias or a true biological asymmetry in susceptibility to pathology.

Understanding the early stages of SD can provide insight into key questions about these syndromes and the functions of the ATLs.^6,26^ How much atrophy is required for symptoms to be apparent? Once semantic deficits can be detected within a single domain, such as person-specific knowledge, how long until deficits occur in other semantic categories? Does impaired social behaviour in right-predominant cases^20,22,27^ precede pre-frontal cortical atrophy? Is the lower proportion of right-lateralized cases primarily a consequence of unrecognized (or unclassified) diagnoses, exacerbated by the clinical overlap with behavioural variant frontotemporal dementia (bvFTD)^27,28^ or asymmetrical hemispheric vulnerability to TDP-43 pathology?^29,30^ Might right-predominant cases have a longer prodromal phase, with greater accumulation of pathology and atrophy before functional impairment?

Here, we consider SD as a nosological entity with a spectrum of lateralization in ATL atrophy.^6,27^ To justify this, we note the shared neuropathology of right- and left-predominant SD,^20,25^ the convergence in clinical phenotype over longitudinal assessment,^31^ and similar distribution of atrophy on imaging^7^ and neurodegeneration^25^ at post-mortem, irrespective of laterality. Including all patients in one study allows unbiased comparison of the earliest symptoms, subsequent clinical and imaging progression and prevalence rates of imaging changes in keeping with SD in a population cohort.

To understand early and presymptomatic SD, we first describe the imaging, clinical and neuropsychological assessments in a case series of SD patients with structural MRI undertaken before recognition of symptoms and a subsequent referral and diagnosis of SD, or who present with incidental imaging findings. We then identify healthy participants with possible SD from the UK Biobank, a prospective UK cohort study of 500 000 participants between 40 and 69 years of age at initial recruitment from 2006 to 2010.^32,33^ Rather than rely on subsequent health records, where coding for SD is potentially limited and of uncertain accuracy, we leveraged the distinctive imaging features of the disease,^7,34^ modifying a methodology we have applied previously in Alzheimer’s disease (AD).^35^ We trained a classifier to differentiate participants with SD from healthy controls and participants with other neurodegenerative diseases, recruited at the Cambridge Centre for Frontotemporal Dementia and the Cambridge Centre for Parkinson-plus. We tested this classifier on a cohort combined from the Neuroimaging in Frontotemporal Dementia^36^ study and the Alzheimer’s Disease Neuroimaging Initiative (ADNI).^37^ We then apply the classifier to the UK Biobank cohort and describe the clinical, cognitive and longitudinal changes of the selected participants with ‘SD-like’ brain imaging.

## Materials and methods

### Participants

#### Cambridge case series

We reviewed all notes of patients with a diagnosis of SD^19,20,38^ who had participated in research studies at the Cambridge Centre for Frontotemporal Dementia. We selected patients who either presented as an incidental finding for imaging performed for a reason unrelated to a dementia or who had clinical or research imaging prior to the onset of their symptoms. Date of symptom onset was determined from clinical records, including history from the patient and informant. We collated longitudinal clinical, neuropsychological and imaging data for these patients. For the first case, detailed neuropsychological testing was performed at initial clinical assessment in a memory clinic and is set out in the Supplementary material. The neuropsychological battery used in the second to fifth cases at the first research visit is described in detail in Rouse *et al*.^27^

#### Participants for model classification and testing

For the Cambridge cohort, we included participants from research studies from the Cambridge Centre for Frontotemporal Dementia, the Cambridge Centre for Parkinson-plus and Cambridge Biomedical Research Centre from 2006 to March 2024 with a 3-T volumetric MRI of suitable quality (see later) with research consent who were either (i) healthy controls; or (ii) had a relevant diagnosis at their most recent clinical assessment. Participants were included in the SD group if they: (i) met clinical diagnostic criteria at their latest clinical assessment for svPPA^19^ or rtvFTD/sbvFTD, applied at the time or in retrospect, noting that during the majority of the ascertainment period the term right SD was in use^20^; or (ii) had confirmed TDP-43 type C pathology at post-mortem; (iii) did not have an alternative more salient clinical diagnosis at latest clinical assessment; (iv) did not have known non-TDP-43 Type C pathology or a genetic mutation causing frontotemporal dementia. We adopted the latter criterion to reduce pathological heterogeneity, recognizing that the clinical syndrome of SD can be caused by other pathologies.^20,23^ Forty-seven participants fulfilled these criteria. The comparator (non-SD) group consisted of 88 healthy controls and 498 participants with other neurodegenerative diseases. The latter group included disorders associated with frontotemporal lobar degeneration or with potential imaging or clinical overlap with SD: behavioural variant frontotemporal dementia (bvFTD),^39^ progressive supranuclear palsy (PSP),^40^ alternative variants of primary progressive aphasia,^19^ primary progressive apraxia of speech,^41^ corticobasal syndrome (CBS),^42^ dementia with Lewy bodies (DLB),^43^ AD and mild cognitive impairment (MCI).^44,45^ Demographic and key clinical characteristics are listed in Table 1 and Supplementary Table 1.

**Table 1:** Demographic and clinical information for the Cambridge cohort

	Semantic dementia	Other neurodegenerative disease	Control	Group statistic	*Post hoc*
Number	47	498	88		–
Scans, *n*	81	498	88	–	–
Diagnoses, *n*	svPPA, 34 sbvFTD, 10 Mixed, 3	AD, 44 bvFTD, 63 CBS, 61 DLB, 42 MCI, 31 lvPPA, 12 nfvPPA/PPAOS, 38 Other PPA, 17 PSP, 190	–	–	–
Sex, female/male	18/29	212/286	43/45	Χ = 1.7, ns	–
Age at baseline imaging, mean (sd)	66.0 (5.7)	71.1 (7.9)	68.2 (7.2)	***F* = 15.5** ***P* < 0.0001**	SD < Other Control < Other
Disease duration at baseline imaging, mean (sd)	4.0 (2.4)	4.3 (2.7)	–	*t* = 0.60, ns	–
Neuropathological primary diagnosis, *n*	TDP-43 C, 7	AD, 16 Synuclein, 3 TDP-43 A/B, 6 CBD, 8 PSP, 61 Pick’s, 4 Tau MAPT, 1 AGD, 1 GGT, 1	–	–	–
ACE-R/ACE-III/100, mean (sd, *n*)	59.4 (17.4, 46)	71.5 (18.9, 492)	94.3 (4.4, 64)	***F* = 60.1** ***P* < 0.0001**	SD < Other, SD < Control, Other < Control
ACE Naming/12, mean (sd, *n*)	3.7 (3.7, 40)	10.5 (2.6, 462)	11.9 (0.4, 54)	***F* = 147** ***P* < 0.0001**	SD < Other, SD < Control, Other < Control
CBI-R, mean (sd, *n*)	62.0 (34.5, 36)	50.3 (32.5 410)	4.6 (3.6, 22)	***F* = 24.7** ***P* < 0.0001**	SD < Control, Other < Control

Clinical scores (ACE-R and CBI-R) are those closest to imaging date. Group differences with *P* < 0.05 are in bold. ACE-R = Addenbrooke’s Cognitive Examination-Revised; AD = Alzheimer’s disease; AGD = argyrophilic grain disease; bvFTD = behavioural variant frontotemporal dementia; CBD = corticobasal degeneration; CBI-R = Cambridge Behavioural Inventory-Revised; CBS = corticobasal syndrome; DLB = dementia with Lewy bodies; GGT = globular glial tauopathy; lvPPA = logopenic variant primary progressive aphasia; MAPT = microtubule-associated protein tau; MCI = mild cognitive impairment; nfvPPA = non-fluent variant primary progressive aphasia; PPA = primary progressive aphasia; PPAOS = primary progressive apraxia of speech; PSP = progressive supranuclear palsy; sbvFTD = semantic behavioural variant frontotemporal dementia; sd = standard deviation; SD = semantic dementia; svPPA = semantic variant primary progressive aphasia; TDP-43 = TAR DNA-binding protein 43.

Our out-of-sample test set for the classifier consisted of participants in the Neuroimaging in Frontotemporal Dementia study (NIFD, also known as the frontotemporal lobar degeneration neuroimaging initiative, http://memory.ucsf.edu/research/studies/nifd)36 and ADNI 3 (adni.loni.usc.edu).^37^ The multicentre NIFD started in 2010. The primary goals of NIFD were to identify neuroimaging modalities and methods of analysis for tracking frontotemporal lobar degeneration and to assess the value of imaging versus other biomarkers in diagnosis. The ADNI was launched in 2003 to test serial biomarkers of diagnosis and progression of mild cognitive impairment and early AD. These datasets in combination include both SD and other neurodegenerative conditions that can cause temporal lobe atrophy. From NIFD, we included baseline imaging for the 127 healthy controls and participants with either svPPA (*n* = 42), bvFTD (*n* = 59) or nfvPPA/progressive non-fluent aphasia (*n* = 37) with imaging data that passed quality control checks. From ADNI 3, we included 276 participants with mild cognitive impairment and 77 participants with AD. We did not include participants with MCI secondary to possible FTD and participants with dementia due to FTD or with primary progressive aphasia, as details were not sufficient to determine the clinical diagnosis required for our machine classifier. Clinical and demographic details for the NIFD and ADNI 3 participants are in Table 2.

**Table 2:** Demographic and clinical information for the NIFD and ADNI cohorts

	NIFD				ADNI	
	svPPA	bvFTD	nfvPPA	Control	MCI	Ad
*n*	42	59	37	127	276	77
Age, mean (sd)	63.1 (5.9)	61.4 (6.8)	68.5 (7.3)	63.4 (7.4)	73.9 (8.1)	75.2 (8.5)
Sex (female/male)	21/21	20/39	20/17	72/55	135/141	34/43
MMSE/30, mean (sd, *n*)	24.9 (4.8, 39)	23.4 (4.6, 59)	25.8 (4.0, 34)	29.4 (0.8, 127)	27.7 (2.1, 275)	22.2 (4.3, 77)
CDR SOB, mean (sd, *n*)	3.9 (2.1, 42)	6.8 (3.3, 59)	2.1 (2.1, 36)	0.05 (0.2, 85)	1.4 (1.1, 273)	5.0 (2.7, 76)

AD = Alzheimer’s disease; ADNI = Alzheimer’s Dementia Neuroimaging Initiative; CDR SOB = Clinical Dementia Rating sum-of-boxes; MCI = mild cognitive impairment; MMSE = Mini-Mental State Examination; nfvPPA = non-fluent variant primary progressive aphasia; NIFD = Neuroimaging In Frontotemporal Dementia; sd = standard deviation; svPPA = semantic variant primary progressive aphasia.

The UK Biobank arm of this study was conducted under application number 20904. Initial recruitment to the UK Biobank occurred between 2006 and 2010, with a subset of participants recalled for imaging assessments. This study uses data from 61 203 imaged participants (mean age 65.0 years, standard deviation 7.7 years, 53% female). At the imaging assessments, participants underwent brain MRI, a questionnaire that included medical history and an assessment of cognitive function. Longitudinal health data were derived from hospital episode statistics, death certification and linked primary health care records, as well as online cognitive assessments performed in 2014 and 2021. We estimate prevalence rates of SD-like imaging changes in this cohort, noting that the mode of recruitment for the UK Biobank led to participants tending to be healthier and of higher socio-economic status than the eligible UK population.^46^

### Imaging acquisition and processing

Details of structural imaging acquisition across the cohorts are in the Supplementary material ‘Methods’ section and Supplementary Table 2. For the Cambridge and NIFD cohorts, we derived volumes for regions of the Desikan-Kiliany Atlas^47^ using Freesurfer^48^ version 6.0.0 on brains that had been skull-stripped using SynthStrip.^49^ Images were reviewed to ensure accurate segmentation, and manual editing was performed where required. Imaging data were of insufficient quality for a small number of participants (Cambridge *n* = 22, Control *n* = 2, AD *n* = 1, MCI *n* = 1, bvFTD *n* = 5, CBS *n* = 4, PSP *n* = 9; NIFD *n* = 3 SD *n* = 1, bvFTD *n* = 2). For ADNI 3 we used the June 2023 UCSF Cross-Sectional Freesurfer 6.0 release,^50^ including only participants who had passed the quality control protocol. For the UK Biobank, regional volumes were derived with Freesurfer 6.0.1 using T1- and T2-weighted imaging where available, with quality control as outlined elsewhere.^51^

We calculated *w*-scores for each region relative to 100 Cambridge healthy controls (mean age: 67.8 years, range 41.4–84.3; 44 female; mean ACE-R: 94.7, range 82–100). For each region, volumes were regressed against age, sex and estimated total intracranial volume. Then, for participants included in the classifier, the difference between measured and predicted volume was divided by the control residual standard deviation. The *w*-scores for the control group then have a mean value of 0 and standard deviation of 1, with +1.96 and −1.96 representing the 2.5th and 97.5th percentiles, respectively.

For the clinical imaging in Case 5 (i.e. the first brain imaging), we used SynthSR^52^ to create a 1 mm isotropic MPRAGE (magnetization-prepared rapid gradient-echo) reconstruction. We subsequently derived volumes for regions of the Desikan-Kiliany Atlas as above. We did not use this imaging in the classifier or longitudinal analysis, given the methodological differences.

### Statistical analyses

Statistical differences across groups for the Cambridge cohort were assessed with the Chi-squared test for categorical data and ANOVA or *t*-test for continuous data (Table 1). For the longitudinal analysis, we used generalized additive models to capture non-linearity in change in *w*-score over time for the Cambridge SD cohort. Models were fitted using the *gam* function from the *mgcv* package^53^ in *R*^54^ version 4.4.1 and the restricted maximum likelihood method, with region *w*-score as the dependent variable, time to onset as the predictor and a random effect per subject. These models were then used to predict *w*-score at onset per individual. Predicted *w*-score at onset for the temporal pole and the ATL (consisting of the temporal pole and middle temporal gyrus) was compared between right-predominant participants and others in a Bayesian model in the R package *brms*. Participants were considered right- or left-predominant depending on the side with the lowest ATL *w*-score. Further details of the Bayesian model testing approach adopted are detailed later.

### Machine classification model hyperparameter tuning, validation and testing

We defined and tuned a random forest classifier on the Cambridge cohort to differentiate participants with SD from other participants. We included grey matter regions of the Desikan-Kiliany Atlas and the lateral ventricles and inferior lateral ventricles. For each region of the atlas, we collapsed across hemispheres by taking the region of minimum *w*-score (i.e. most atrophied) of the left and right comparative region, except for the ventricles, where we took the maximum *w*-score. This was to ensure that selection in the UK Biobank was not biased by a greater number of left-predominant participants in the training datasets. We also included features of left-right difference across hemispheres, and three further derived features: the region with minimum *w*-score; minimum *w*-score difference from non-temporal cortical regions to the temporal pole; and minimum *w*-score difference from non-temporal cortical regions to the middle temporal gyrus.

Model training, tuning and testing were performed using Scikit-learn^55^ in Python. We chose random forests,^56^ an ensemble-based classification method built on decision trees, due to its robustness to overfitting, an important consideration given the moderate training and test dataset sizes. Given that our training and test sets were imbalanced, we focused on the F1 score for the SD class, which is the harmonic mean of precision and recall. We performed a grid search with maximum tree depth ranging from four to eight, a varying number of estimators (50, 100, 200, 300, 400, 500) and across different thresholds with 100 repeats of 5-fold cross-validation. The model was then re-fitted on the full Cambridge dataset with the selected parameters and threshold and taken for out-of-sample testing on the combined NIFD and ADNI 3 datasets. We defined a lower secondary threshold with grid search parameters selected to maximize the F2 score, which weights towards recall over precision. We additionally calculated permutation importance on the test set. Given that most participants with SD had left-predominant atrophy, we repeated the model fitting with an adjusted sample weighting of 2 for those with right-predominant SD and 1 for all other participants.

### Anterior temporal lobe atrophy in the UK Biobank

#### Participant selection

A random forest model fitted on the whole cohort with the selected parameters was then applied to the UK Biobank cohort. We reviewed images for participants above the primary threshold to ensure that there was no non-neurodegenerative structural pathology (e.g. arachnoid cysts, infarcts) impacting the assessment of regional volume in the ATLs. Remaining participants above the threshold were considered to be ‘SD-like’. We divided them into right-predominant and left-predominant groups depending on the side of lowest ATL *w*-score. We calculated population prevalence adjusted for the UK population aged 45–85 as per the 2011 UK-wide census.^57^ We assessed evidence for or against differences in polygenic risk scores for AD.^58^ We performed secondary analyses at the lower threshold determined earlier for a model with up-weighting of participants with right-predominant SD, and for the machine learning model defined earlier but without left-right differences included as features.

#### Cognitive testing and self-reported health

At the imaging visit, UK Biobank participants underwent a touchscreen assessment of cognitive function and a questionnaire. They were asked to detail their medical history and to rate their general health. For the cognitive assessment, participants completed a battery of up to 11 assessments covering multiple cognitive domains.^32,59^ These included tests of visual and verbal memory (a prospective memory task, pairs matching, paired associate learning), tests of verbal and non-verbal reasoning (fluid intelligence test, matrix pattern completion), tests of executive function (trail making, symbol digit substitution, tower arranging), reaction time and forward digit span. Of particular interest here was the picture vocabulary task, which used an adapted staircase design to assess participants’ ability to match words to sets of four pictures. This was adapted from the National Institute of Health Picture Vocabulary Toolbox but with incomplete modification of US English, resulting in a poorly calibrated sampling algorithm for UK participants. We therefore followed Giunchiglia and colleagues^60^ to derive a score of specific naming ability. For the alphanumeric trail making task, no maximum time limit was applied, with no information available for individuals who abandoned the task on time spent or answers attempted. Therefore, for individuals at the imaging assessment or the 2021 online follow-up who abandoned the task, we used multivariate imputation by chained equations through the *mice* package in R,^61^ based on numeric trail making, digit symbol, fluid intelligence and demographic information (40 imputations, predictive mean matching).

We used a Bayesian approach to assess for differences in cognitive function between the SD-like group and other UK Biobank participants. Bayesian testing was performed in *brms* in R with 2000 iterations and four chains. We used weakly informative priors for coefficients,^62^ with choice of distribution family and model prior (Supplementary Table 3). Age, sex and highest qualification were included as covariates of no interest, with the latter converted to years of education as per the International Standard Classification for Education coding.^63,64^ Hypothesis testing used the *hypothesis* function in *brms*, testing a hypothesis of no difference between groups, with Bayes factors derived via the Savage-Dickey density ratio.^65^ We also report statistics for comparable frequentist tests in the Supplementary material.

#### Longitudinal and health outcome data

Outcome data are available for UK Biobank participants, including hospital episode statistics, incorporating discharge destination following inpatient admission, death certification, self-reported data, and data from primary care records for a proportion of participants until 2017. Participants were also asked to complete online health ratings in 2020 and 2023 and online cognitive follow-up in 2014 and 2021. These included questions of whether participants had difficulties with thinking or with communication. The cognitive assessment battery at the 2021 iteration consisted of trail making, fluid intelligence, matrix pattern completion and symbol digit substitution. We compared results for participants above the primary threshold, with an initial imaging visit before or during 2021, with other UK Biobank participants at the later online follow-up visit. We assessed longitudinal change in overall health rating and fluid intelligence, selected as repeat assessments in these domains had been performed since baseline recruitment. We standardized scores relative to the Biobank population at each time point. We used mixed linear effect models including a group-by-time interaction, age as a covariate of no interest, and subject as a random effect using the lme4 package in *R*.^66^

## Results

### Early and presymptomatic semantic dementia: a case series

**Figure 1:**
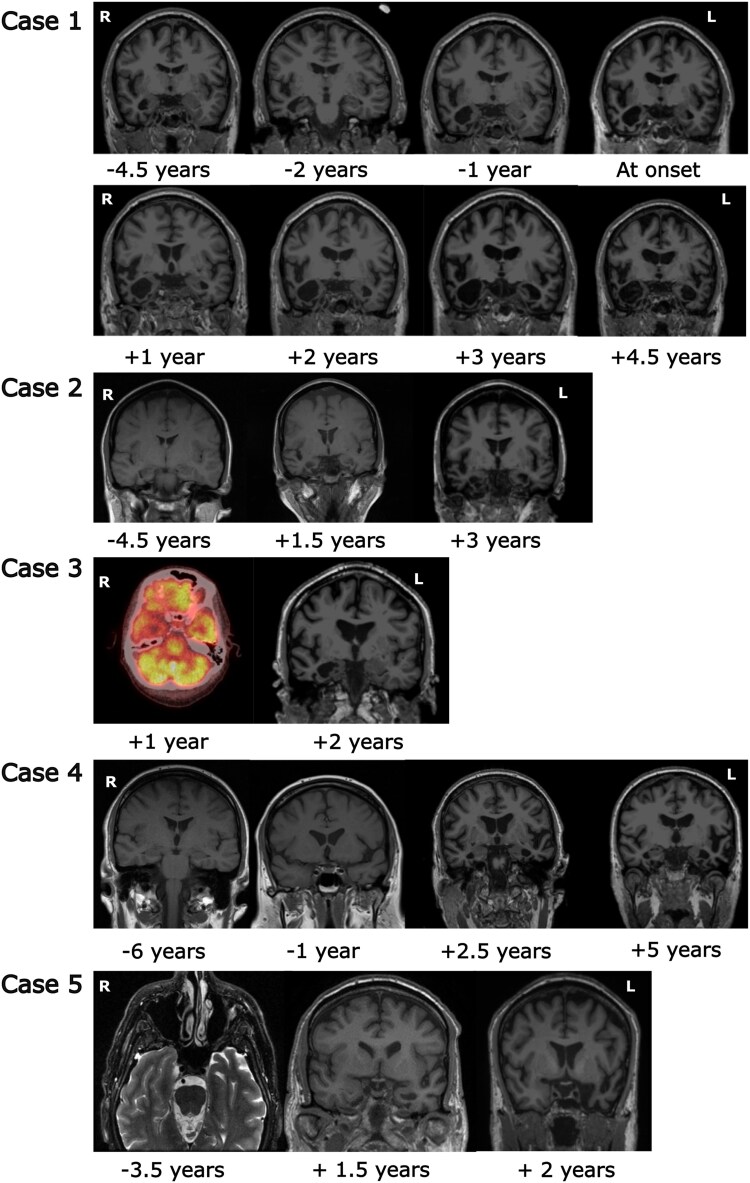
**A case series of early and presymptomatic imaging in semantic dementia.** Patients with imaging performed either in the presymptomatic phase or detected as an incidental finding. Imaging is shown in radiological convention. The text below each image is the time to or from symptom onset as determined by the earliest date with symptoms according to the patient or their informant. L = left; R = right.

We identified five people who developed SD with presymptomatic or early MRI. Figure 1 and Table 3 outline the imaging changes and key neurocognitive testing, with further details of the cases included in the Supplementary material ‘Results’ section, Supplementary Fig. 1 and Supplementary Tables 4 and 5. Two patients were involved as healthy volunteers in research studies with an imaging component (Cases 1 and 5); two had brain imaging as part of investigations for cancer (Cases 3 and 5); and two had imaging for neurological indications unlikely to be related to their dementia (Cases 2 and 4). In Case 1, there is post-mortem confirmation of TDP-43 type C pathology.

**Table 3:** Summary of clinical information, indicative imaging findings and cognitive testing for images in case series shown in Fig. 1

Case	Time to symptom onset (years)	Time to diagnosis (years)	Indication	Imaging (*w*-score)	Cognitive testing
1	−4.5	−7.5	Research study healthy volunteer	R temp pole −2.3 R inf lat vent 3.7	–
	−2	−5	Research study healthy volunteer	R temp pole −3.9 R inf lat vent 17.9	–
	−1	−4	Research study healthy volunteer	R parahipp. −3.6 R pars orbital −1.8	ACE-R 99/100, MMSE 30, BDI 3
	At onset	−3	Research study healthy volunteer	R temp pole −4.6 R sup temp −2.5	MOCA 29, AES-C 37/68, CBI 15, AES-S 39, NPI 7
	+1	−2	Research study healthy volunteer	L accumbens −2.5 L inf lat vent 2.7	Paired associate learning task—above group mean
	+2	−1	Research study healthy volunteer	L temp pole −0.8 R inf lat vent 29	ACE-R 94, MMSE 29, BDI 3, MOCA 29
	+3	At diagnosis	FTLD study	L putamen −2.3 R lat orbital −2.4	ACE-R 96, **GNT 10**, MMSE 30, **CBI-R 86**
	+4.5	+1.5	FTLD study	R caudate −2.9, R lat occipital −1.5	**ACE-R 77**, MMSE 28, **CBI-R 64**
2	−4.5	−6.5	Transient sensory symptoms	Mild R ATL atrophy	–
	+1.5	−0.5	Chronic headache	–	**ACE-III 64, CBI-R 67**
	+3	+1	FTLD study	R temp pole −5.0 L temp pole −3.5	**ACE-R 61, BNT 8,** MMSE 25, **CCT 17, CBI-R 111**
3	+1	−1	Tonsillar cancer	R temp hypometabolism	–
	+2	At diagnosis	FTLD study	R temp pole −4.0 R inf lat vent 13.4	ACE-R 92, MMSE 30, **CBI-R 35**, BNT 30, CCT 31, **LNM 31, FFM 19**, CN 32
4	−6	−7 (MCI)	Collapse	No visible atrophy	–
	−1	−2 (MCI)	Lower limb paraesthesia	Mild atrophy L ATL	–
	+2.5	+1.5 (MCI)	MCI study	L temp pole −3.9 L mid temp −4	**ACE-R 77,** MMSE 27, MBI 0
	+5	+4 (MCI)	FTLD study	R temp pole −2.3 R mid temp −5.3	**ACE-R 63, BNT 11**, MMSE 27**, CCT 16**, CBI-R 12
5	−3.5	−5	Parotid tumour	ATL asymmetry L temp pole −1.4	–
	+1.5	−0.1	Research study healthy volunteer	–	–
	+2	+0.3	FTLD study	L temp pole −3.4 L inf lat vent 6.2	**ACE-R 75**, MMSE 29, CBI-R 6, **BNT 9, CCT 21**

Regions listed are those of the Desikan-Kiliany Atlas. Symptom onset is the earliest date with symptoms according to the patient or their informant. Time to diagnosis is of any neurodegenerative disease. Cognitive tests in bold represent scores that deviate from recognized cut-off data or that significantly differ from control data as set out in Rouse *et al*.^27^ ACE-R = Addenbrooke’s Cognitive Examination-Revised; AES = Apathy Evaluation Scale; ATL = anterior temporal lobe; BDI = Beck Depression Inventory; BNT = Boston Naming Test; CBI-R = Cambridge Behavioural Inventory-Revised; CCT = Camel and Cactus Test; CN = Cambridge Naming Test; FTLD = frontotemporal lobar dementia; FFM = Familiar faces matching; GNT = Graded Naming Test; L = left; LNM = Landmark-Name Matching; MBI = Mild Behavioural Inventory Checklist; MCI = mild cognitive impairment; MMSE = Mini-Mental State Examination; MOCA = Montreal Cognitive Assessment; NPI = Neuropsychiatric Inventory; R = right.

These cases highlight clinically detectable atrophy present 3–5 years prior to the recognition of symptoms by the patient or relatives and other informants (e.g. Cases 1 and 5). Particularly pronounced atrophy with minimal or no symptoms was observed in Cases 1 and 3, with asymmetric right-sided predominant atrophy. The case series also demonstrates the variability in the degree of atrophy at symptom onset, with Case 4 reporting symptoms within a year of imaging but showing only minimal left anterior lobe atrophy.

In Cases 1 and 3, the patients performed well on global cognitive testing screening in the early and presymptomatic phases of the disease. In these right-predominant cases, detailed testing revealed a semantic impairment, which was not selective to social-semantic knowledge or familiar face recognition. In Case 3, this included impairment in a synonym judgement task^24^ and a landmark-to-name matching task.^67^ These deficits occurred at most 2 years after the onset of behavioural symptoms. The behavioural changes were mild, and in Case 3 considered not sufficient to justify seeking medical attention or impacting employment.

In contrast, in Cases 4 and 5 with left-sided predominant atrophy, there were early deficits in global cognitive screening tests. This was despite minimal functional impairment, with poor performance on tests of verbal and non-verbal semantic knowledge, including the Camel and Cactus Test,^18^ the Boston Naming Test,^68^ the synonym judgement task and landmark-to-name matching task. Across all cases, there were variable deficits in tests of executive function in the early stages of disease (Supplementary Tables 4 and 5).

### The Cambridge semantic dementia cohort

**Figure 2:**
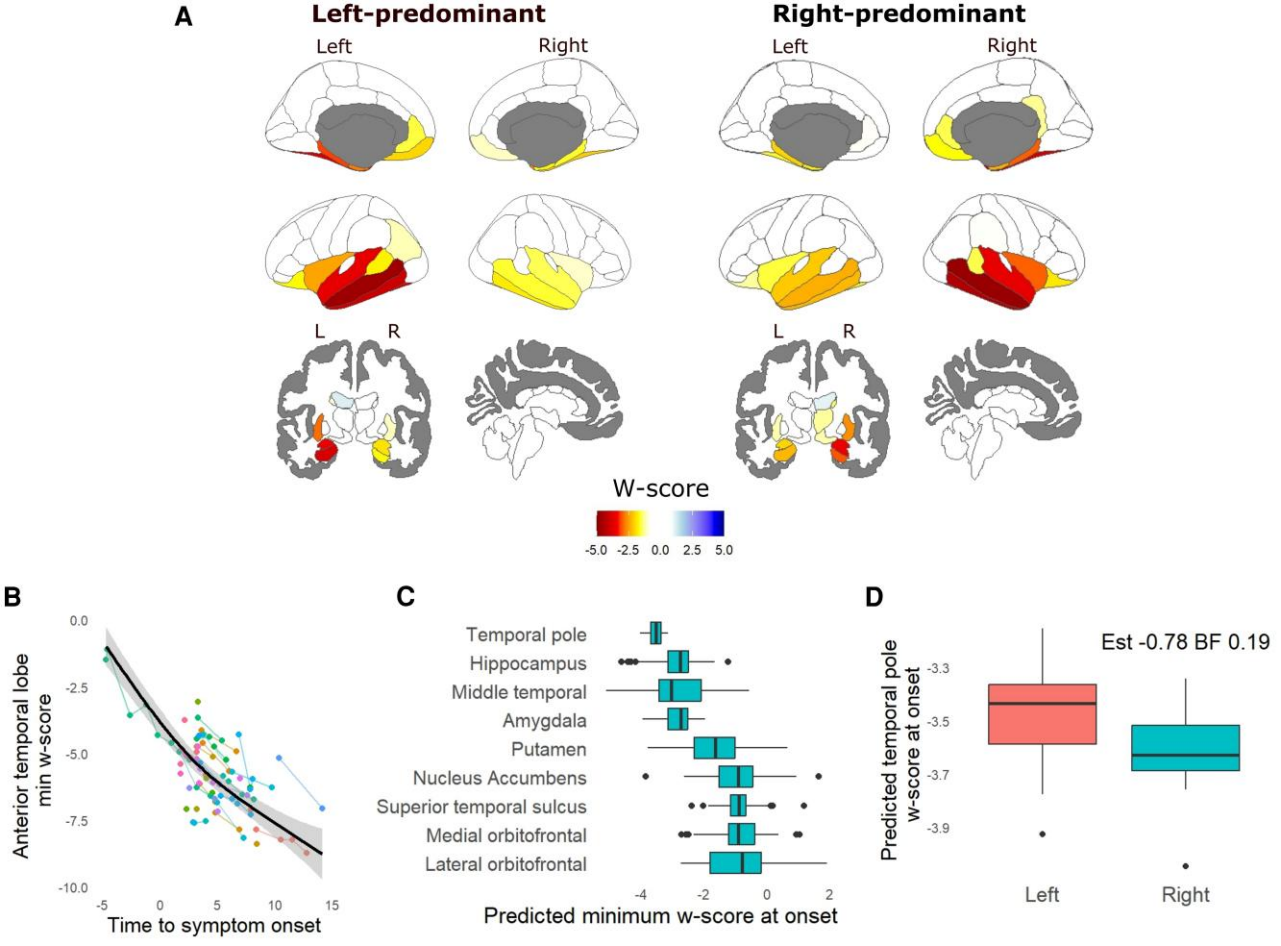
**The Cambridge semantic dementia cohort.** (**A**) Atrophy patterns for the Desikan-Kiliany Atlas for the baseline research scan for participants from the Cambridge semantic dementia cohort, with *w*-scores relative to 100 controls. (**B**) Generalized additive mixed model of anterior temporal lobe (temporal pole + middle temporal gyrus) *w*-score against time to onset. (**C**) Predicted minimum *w*-score at onset for selected regions across hemispheres. (**D**) Moderate evidence for greater predicted temporal pole atrophy at onset in those with right-predominant symptoms at onset. BF = Bayes factor.

Atrophy patterns for the Cambridge SD cohort at baseline research scan are shown in Fig. 2A, with prominent ATL atrophy and relative sparing of the frontal, parietal and occipital lobes. We used generalized additive models to capture non-linearity of progression of peak ATL atrophy and at other key structures over time (Fig. 2B and C). This estimated an average *w*-score at onset at the ATL of −3.9. Participants with SD were within the 2.5th control percentile for this region from 3.2 years prior to onset. There was moderate evidence for greater atrophy at onset in the right-predominant cases when considering the temporal pole [Fig. 2D; Beta −0.78 confidence interval (CI) −1.43 to −0.12 Bayes factor (BF) 0.19], with anecdotal evidence for no difference for the whole ATL (Beta −0.22 CI −0.91 to 0.49 BF 2.33).

### Model parameter tuning, validation, and out-of-sample testing

We proceeded to define and test a classifier to distinguish participants with SD from healthy controls and people with neurodegenerative conditions. Repeated cross-validation for the Cambridge dataset for the highest performing model parameters (threshold 0.44, maximum depth 4, number of estimators 100) produced accuracy: 0.98, recall: 0.92, precision: 0.83 and F1 score: 0.87 (Supplementary Table 6). Variables with the highest feature importance (Supplementary Table 7) reflect atrophy in the ATL and *w*-score difference from the ATL to non-temporal cortical regions. Of the ten individuals with a final non-SD diagnosis labelled at the primary model threshold as in the SD group, seven had a diagnosis of bvFTD, two Ad and one PSP. We calculated an alternative threshold maximizing the F2 score (optimizing for recall) with maximum depth and number of estimators as per the primary threshold. We repeated model testing with features measuring cross-hemisphere volume difference removed, which resulted in deterioration in model performance. The performance metrics for these adjusted models are listed in Supplementary Tables 8 and 9.

The model performed well in the combined NIFD and ADNI test dataset (Supplementary Table 6; accuracy: 0.99, recall: 0.88, precision: 0.95, F1 score: 0.91). Of the 576 individuals with non-SD diagnoses, only two were assigned to the SD group (bvFTD *n* = 1, AD *n* = 1). Thirty-seven of the 42 SD patients were correctly identified. We therefore proceeded to test for the presence of individuals with imaging changes in keeping with SD in the UK Biobank.

### Anterior temporal lobe atrophy in the UK Biobank

**Figure 3:**
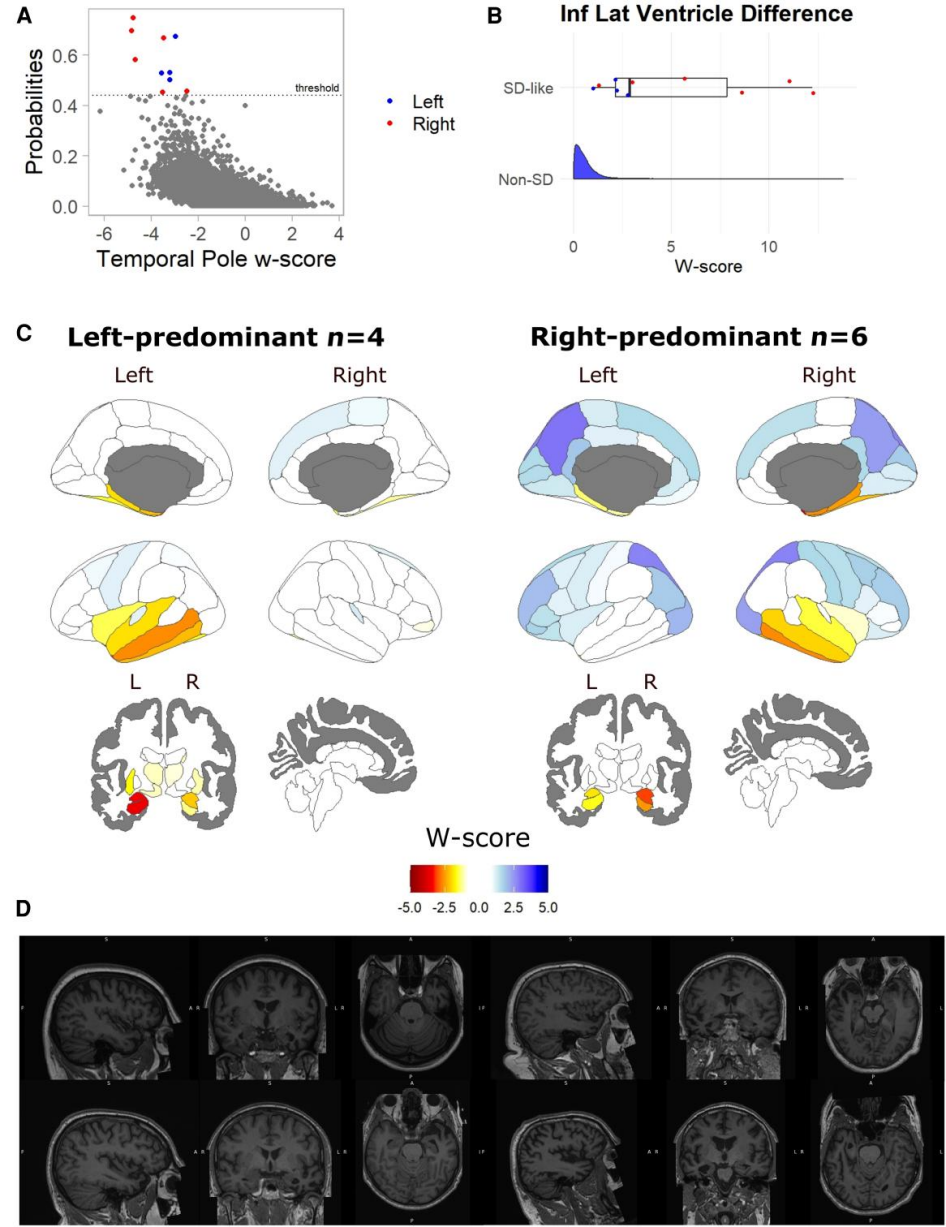
**Participants with semantic dementia-like imaging in the UK biobank.** (**A**) Derived probability from the final random forests model fitted in the UK Biobank, with the primary threshold shown with the dashed line. (**B**) Difference between left and right inferior lateral ventricle *w*-score for the selected participants. (**C**) *W*-score by regions of the Desikan-Kiliany Atlas for the UK Biobank selected participants. (**D**) T1-weighted MRI in radiological convention for sample semantic dementia-like UK Biobank participants, with further imaging shown in Supplementary Figs 2 and 3, reproduced by kind permission of UK Biobank^©^.

The model probability for 15 participants in the UK Biobank exceeded the primary threshold (Fig. 3A–D). Five participants were excluded due to non-neurodegenerative structural pathology in the temporal lobes. The median age of the remaining 10 individuals at the imaging visit was 64.5 (range 55–73, standard deviation 5.7). All 10 were right-handed. Six had predominantly right ATL atrophy, leading to an estimated population prevalence between ages 45 and 85 of 5.9/100,000, and four had left-sided predominant atrophy (estimated prevalence 4.8/100 000). ATL atrophy was particularly marked in some individuals with right-sided predominant changes (Fig. 3A and B; T1 MRI shown in Fig. 3D and Supplementary Figs 2 and 3). The selected individuals showed significant ATL asymmetry, demonstrated by a difference between hemispheres in inferior lateral ventricle *w*-scores (Fig. 3B). There was moderate evidence for no difference from the UK Biobank population in polygenic risk score for AD (Beta −0.05, CI −0.65 to 0.58, BF 3.12).

Eighteen individuals had model probabilities above the lower threshold (right-predominant *n* = 13, left-predominant *n* = 5). Twenty individuals had model probabilities above the threshold using a model without features of left-right difference (right-predominant *n* = 15, left-predominant *n* = 5). Applying the model with adjusted weighting to preference those with right-predominant atrophy, we found that 9 of the 10 participants in the primary cohort were again classified as ‘SD-like’. A further five participants were also above the threshold, all with right-predominant or symmetrical atrophy patterns. Three of these participants were included when considering the lower threshold. Model probability for these additional five participants was above the threshold but below the nine participants included in the main model.

#### Neuropsychological profile

**Figure 4:**
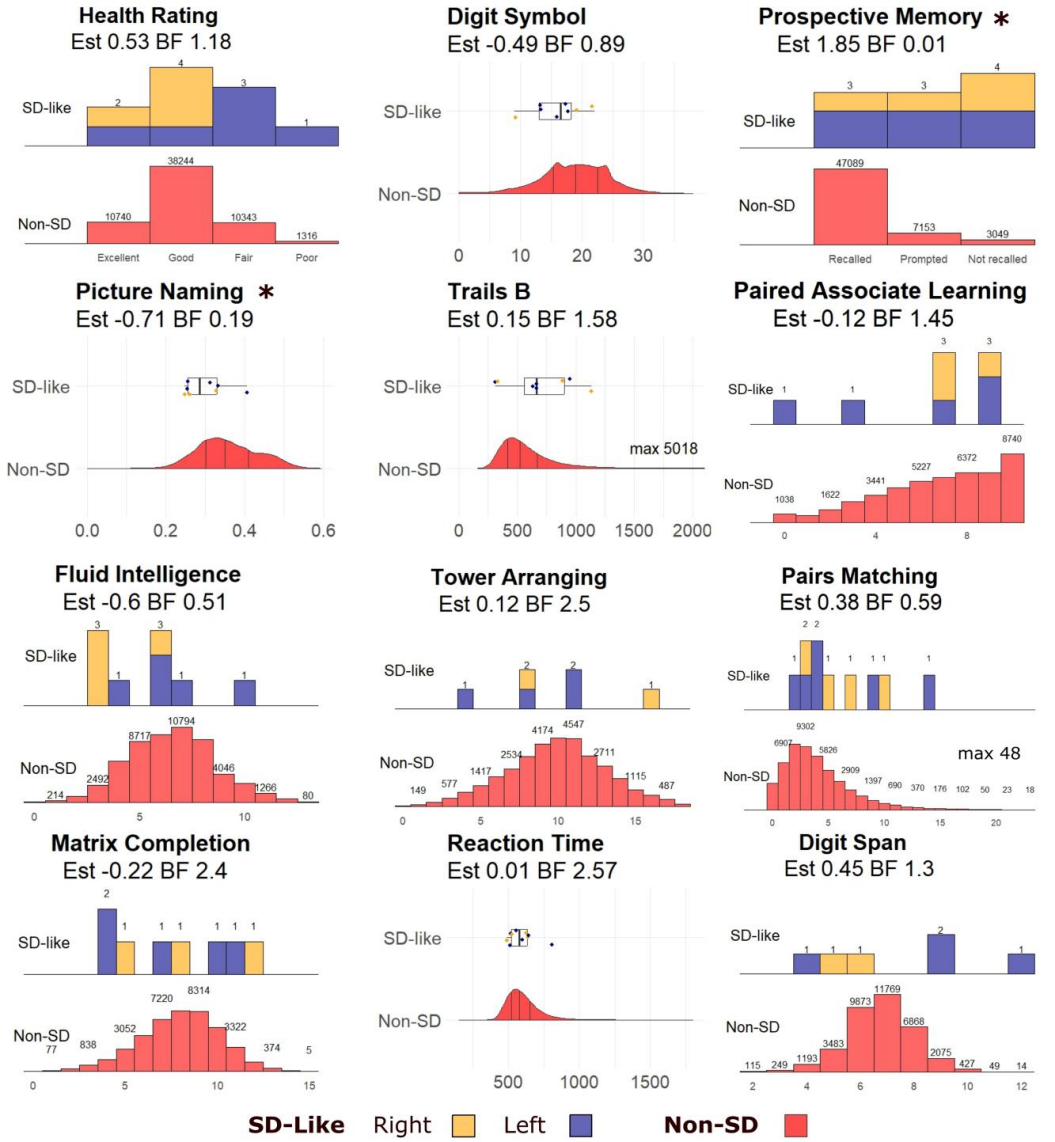
**Health rating responses and cognitive testing for the selected participants at the imaging assessment.** For each tests scores for the selected participants are shown above, with the distribution for the rest of the UK Biobank participants within the imaging arm *below*. There was at least moderate evidence for deficits in prospective memory and picture naming, denoted with an asterisk. SD-like participants with right-predominant atrophy in dark blue and with left-predominant atrophy in orange. BF = Bayes factor; SD = semantic dementia.

The cognitive testing profile from the UK Biobank imaging visit is shown in Fig. 4 and in Supplementary Table 10. There was moderate evidence for lower scores in the 10 selected individuals in a picture naming task (Beta −0.71, CI −1.32 to −0.13, BF 0.19). There was very strong evidence for poorer performance on a prospective memory task (Beta 1.85, CI 0.79–2.85, BF 0.01), in which participants were required to select an orange circle when presented with a prompt to select a blue square, having been given the instruction earlier in the assessment. For other cognitive tests, evidence for or against a difference was at anecdotal levels. There were lower fluid intelligence scores in individuals with left-sided predominant atrophy (left-sided predominant Beta −1.21, CI −2.05 to −0.37, BF 0.05; right-sided predominant Beta −0.01, CI −0.76 to 0.75, BF 2.57).

**Figure 5:**
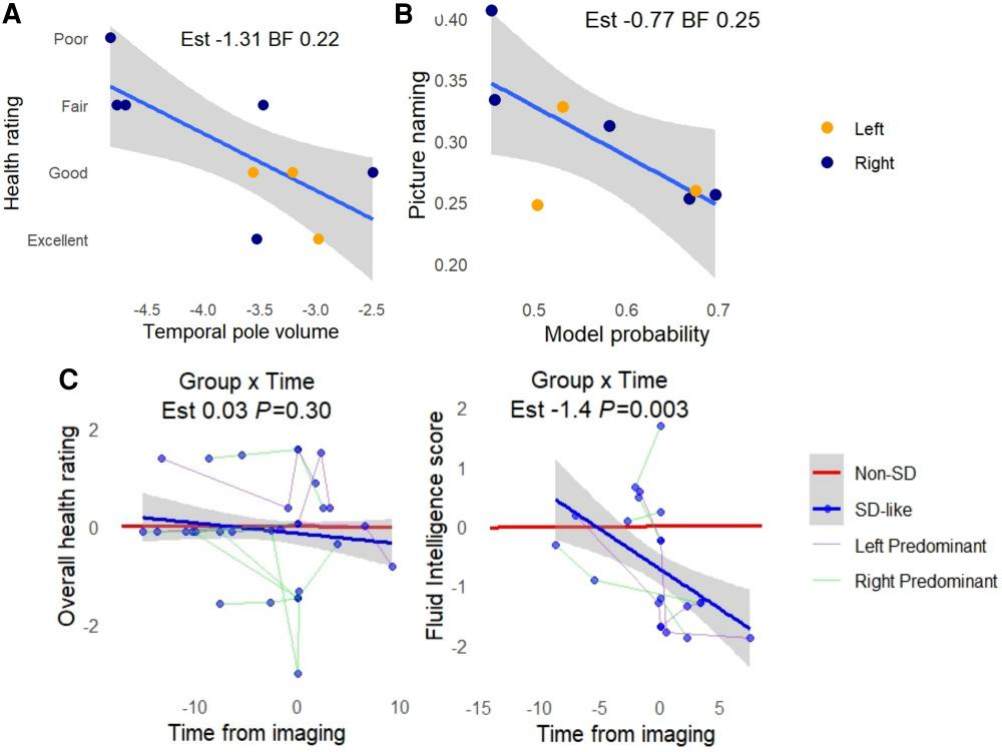
**Cognitive assessment, atrophy and model probability at imaging assessment and longitudinally.** (**A**) More severe atrophy of the temporal pole is associated with poorer health rating for those selected as SD-like from the UK Biobank resource. (**B**) Model probability is related to picture naming for selected SD-like participants. (**C**) Those with SD-like imaging showed longitudinal decline in fluid intelligence, but not in overall health rating. Test scores are standardized, with more negative numbers meaning poorer scores/worse health rating. BF = Bayes factor; SD = semantic dementia.

Greater atrophy at the temporal pole was associated with poorer reported global health (Beta −1.31, CI −2.67 to 0.04, BF 0.22; Fig. 5A). Higher model probability for the selected participants was associated with poorer performance in the picture naming task (Beta −0.77, CI −1.38 to 0.03, BF 0.25; Fig. 5B), with anecdotal evidence against a relationship with fluid intelligence (Beta 0.07, CI −0.89 to 1.05, BF 2.3) and prospective memory (Beta −0.06, CI −1.12 to 1, BF 1.79).

Results of cognitive testing for participants selected using a model without cross-hemispheric differences are in Supplementary Table 11. As in the primary group, there were deficits in picture naming and prospective memory, together with moderate evidence for poorer fluid intelligence and reported global health. The cognitive profile for the participants selected at the lower threshold (Supplementary Table 12) showed moderate or stronger evidence of deficits in picture naming, fluid intelligence, prospective memory, digit symbol and paired associate learning.

#### Longitudinal follow-up data

The outcome data from health records for UK Biobank participants are substantial but incomplete, with primary health care data currently provided until 2017. No participants had a recorded diagnosis of dementia from self-report, hospital episode statistics or death certification. One of the 10 selected individuals with predominantly right-sided atrophy died approximately 4 years post-imaging with motor neuron disease. In the post-imaging period, this participant had also been newly coded as having a developmental disorder of speech, with no further information provided. Another participant died from cancer within 2 years of imaging. Five participants had at least one inpatient hospital admission after imaging acquisition. None were coded as being discharged to a nursing or care home.

Imaging for 7 of the 10 was acquired in the year of, or prior to, the most recent scheduled online follow-up in 2021. This was completed by five of these seven. Median time from imaging to online assessment was 3.3 years (standard deviation 2.8 years). Participants were asked if they had communication difficulties; two stated that they did, one did not know, and two did not provide a response. When asked if they had difficulties thinking, one responded that they did not, one that they did, one did not know, and two provided no response. For the five individuals there was very strong evidence for a deficit in fluid intelligence (Beta −1.3, CI −2.08 to −0.55, BF 0.01), moderate evidence for deficit in the alphanumeric trails task (Beta 0.26, CI 0.03–0.49, BF 0.23) and in matrix pattern completion (Beta −0.87, CI −1.71 to −0.03, BF 0.32), and anecdotal evidence against a difference in performance in digit symbol (Beta −0.13, CI −0.93–0.7, BF 2.34).

We additionally assessed longitudinal models for fluid intelligence and global health rating (Fig. 5C) where multiple repeat assessments since recruitment had been performed. We found that time from imaging visit was associated with poorer scores in fluid intelligence for the SD-like group (Group × Time interaction: Beta −1.4, *P* = 0.003) but not with worse overall health rating (Group × Time interaction: Beta 0.03, *P* = 0.30).

## Discussion

Our study confirms and characterizes the proposed presymptomatic stage of SD, leveraging the power of large open-access datasets. In our case series, we showed that atrophy can clearly be present 3 to 5 years before patients or their informants recognize symptom onset in right- and left-predominant cases. The imaging measures reliably and accurately differentiated clinical SD from other neurodegenerative diseases, including other syndromes causing frontotemporal lobar degeneration, and healthy controls. We found apparently healthy individuals with striking ATL atrophy in a pattern consistent with SD, who had subtle deficits on cognitive testing suggestive of semantic impairment and longitudinal deterioration in other cognitive domains. Using the clinically unbiased ascertainment of the UK Biobank, we showed that right-predominant cases are at least as common as left-predominant cases. However, the projected atrophy at symptom onset was greater in right- than left-predominant cases, in keeping with the difference in clinical prevalence between left- and right-SD.

Significant atrophy developing several years before symptom onset has been observed in genetic neurodegenerative conditions^69,70^ and sporadic AD. We found the degree of focal atrophy in our presymptomatic SD and ‘SD-like’ cases to be more marked. Establishing symptom onset in neurodegeneration is challenging, a factor in the drive towards biomarker based diagnostic criteria in Alzheimer’s disease^44^ and Parkinson’s disease.^71^ As in our case series, left- and right-lateralized SD can present with prolonged subtle prodrome.

Interpretation of these symptoms by patients, family members, other informants and healthcare professionals influences when the disease is considered to have started. We found that more marked atrophy of the temporal pole was associated with poorer reported general health.

We found evidence in the right-lateralized cases (Cases 1 and 3) that semantic deficits were not selective to social concepts and may be accompanied by minimal behavioural disturbance. In the fifth case, where left-predominant atrophy was detected following participation as a healthy control in a research study, there were semantic deficits in multiple domains including social-semantic knowledge. These findings support the conceptualization of SD as defined by generalized degradation of conceptual knowledge, following atrophy to a transmodal functionally-unitary hub underpinned by the ATLs.^6,26,27,72,73^ This framework is challenged by the fact that people with right-predominant ATL atrophy often present with impaired social behaviour. We have argued^26,27^ that this can be accounted for by evidence from other case series that right-predominant SD have both greater overall ATL atrophy and greater atrophy beyond the ATLs.^6,31,74^ Our Case 1 supports this, given that the timing of symptom onset and reported behavioural disturbance coincide in time with atrophy beyond the right ATL.

Among participants from the UK Biobank selected by the classifier as ‘SD-like’, we found picture-naming deficits consistent with semantic impairment, and these deficits correlated with the model's probability. These individuals did not self-report any dementia at the imaging assessment. We also found deficits in a prospective memory task. This one-item task requires response inhibition from participants, and correlates only weakly with markers of everyday memory function but more strongly with measures of executive function and reasoning.^59^ Evidence for or against involvement of other cognitive domains at the imaging assessment was equivocal, with deterioration in tests of executive function and reasoning in longitudinal follow-up. There was no biomarker confirmation available that these participants did not have AD, although the pattern of atrophy would be unusual (as shown by the classifier), particularly in individuals with minimal symptoms, and the pattern of cognitive deficits differed from that observed for AD in subsequent health record data^75^ or as having typical AD imaging changes.^35^ There was evidence against any difference in the AD polygenic risk score. The single participant of the selected 10 with a coded neurodegenerative diagnosis in longitudinal follow-up, who had right-predominant atrophy, had motor neuron disease, and therefore likely TDP-43 neuropathology.^20,76^

We found prevalence rates of presymptomatic SD-like imaging to be higher than reported rates of symptomatic disease in the UK in some studies,^10,77^ noting the variation in incidence observed across regions.^78^ This was particularly the case for right SD, despite the fact that our training set had a majority of left-predominant cases. We emphasize two key considerations. First, there is likely under-recognition and reporting of this syndrome, given the clinical challenges in differentiating right SD from other syndromes associated with frontotemporal lobar degeneration,^20,79^ with diagnostic criteria for sbvFTD/rtvFTD only recently proposed.^20,21^ Second, computational models of ATL atrophy and SD^80-82^ suggest bilateral damage is required for marked semantic impairment, with some domains of processing asymmetrically supported (e.g. naming), meaning that left-lateralized atrophy leads to increased levels of anomia compared to right-lateralized. A corollary of these models is that people with right-lateralized SD would then have a longer period of neuropathological accumulation on average before symptoms manifest. We note four findings that lend support to this: (i) Case 1, where the period with detectable atrophy in right-predominant SD was longer than duration from diagnosis to death; (ii) Case 3, where significant atrophy was associated with only mild impairment on a detailed neuropsychological battery; (iii) the selected cases with the most marked ATL atrophy in the UK Biobank had right-predominant atrophy; and (iv) the right-lateralized cases in the Cambridge cohort had greater temporal polar atrophy at symptom onset. Novel neuropsychological tests may be more sensitive to the early right-lateralized SD, preceding functional impairment.

Our findings have clinical implications. With the rise of brain health clinics^83,84^ and the increased use of imaging for indications unrelated to neurodegeneration, the chance of incidental ATL atrophy is increased. Prognostication in these cases is challenging; we find that such patients do progress but may remain independent for many years. This cohort is a potential target for clinical trials,^85^ given that atrophy can be identified with relatively minimal symptoms and maintained daily function. Studying patients with marked but highly focal atrophy provides an opportunity to understand factors that determine progression and disease spread in a sporadic neurodegenerative disease. We were able to accurately differentiate participants with SD from those with other neurodegenerative diseases, including in a held-out test set, with similar precision to another study using deformation-based morphometry to classify FTD variants in the NIFD dataset.^86^ It is plausible that these methodologies could be refined to provide a clinically useful imaging-based SD classifier for patients presenting with neurodegenerative diseases.^87^

Our study has limitations. The model was trained on participants with predominantly established disease and therefore may be less sensitive to very early atrophy. We included features capturing asymmetry in our training model to increase capture of individuals with highly focal ATL atrophy. SD shows distinct patterns of atrophy^7,8^ with variation in asymmetry and degree of frontal lobe involvement, and the model gives greater probabilities to individuals with characteristic atrophy distribution. We therefore risk missing participants with SD or underlying TDP-43 type C neuropathology with atypical topographical disease. The UK Biobank provides a broad cognitive battery not refined to capture all deficits in SD, although it includes a relevant naming task. Limited conclusions can be drawn about behavioural and social cognitive changes in these participants, with no informant history provided. There is only partial available longitudinal health record data, and even were this to be provided, coding of primary progressive aphasia and behavioural disturbance are unlikely to be reliable. For three subjects from our case series, only clinical-grade structural imaging was available prior to symptom onset. Therefore, more subtle atrophy may be missed. In the case series and our machine learning model, we have neuropathological confirmation in only a proportion of cases. SD has the advantage of high clinicopathological correlation^20,23^ and similarity in patterns of atrophy between left- and right-lateralized cases.^25^

To conclude, we find that individuals with SD have significant atrophy prior to symptom onset. Despite ATL atrophy, there can be strong performance in screening tests of global cognition, although with deficits in semantic domains. Greater atrophy is associated with poorer health ratings, with progression to involve wider cognition over years. In contrast to clinical series of prevalent cases, our prospective analysis suggests that the prevalence of left and right-predominant atrophy is similar. We show the benefits of considering SD, whether left- or right-lateralized, as a single disorder defined in part by imaging features, with a continuous spectrum in the degree of lateralization in atrophy.

## Supplementary Material

awaf351_Supplementary_Data

## Data Availability

Data used in this study from ADNI, NIFD and the UK Biobank are publicly available following the relevant application processes. For neuroimaging and clinical data for the Cambridge cohort, patients’ consent does not permit open data sharing, but managed access is permitted. Requests for data sharing from affiliated researchers should be made to the senior author and will likely require a Material Transfer Agreement to protect confidentiality and restrict commercial exploitation.
