# Supplementary material for: The presymptomatic and early manifestations of semantic dementia
Source: Brain. 2025 Sep 23;149(5):1663–78. doi: 10.1093/brain/awaf351 (PMC13140614; doi:10.1093/brain/awaf351)
Supplement: awaf351_Supplementary_Data [file awaf351_supplementary_data.pdf]

Supplementary Materials

Supplementary Methods

Research images for the Cambridge cohort were all acquired between 2006 and 2024 at the Wolfson Brain Imaging Centre or the MRC Cognition and Brain Sciences Unit. A 3-Tesla high resolution T1-weighted structural image was obtained (slice thickness 1-1.25mm, median TE 2.93ms, median TR 2.3, median TI 0.85, flip angle 8-12°, with further details in Supplementary Table 2). For participants with SD, we collated longitudinal 3-Tesla T1-weighted structural imaging acquired at the same centres.

Images used from the NIFD cohort in this study were volumetric magnetization-prepared rapid gradient echo (MPRAGE) sequences of the whole brain at 3-Tesla (1mm isotropic; matrix = 240 × 256; TR = 2.3s; TE = 3 ms; TI 900 ms; flip angle = 9°). The ADNI 3 imaging protocol included a 3-Tesla volumetric MPRAGE which we used in this study (voxel size 1mm isotropic, 208x240x256 acquisition, TR 2.3s, minimum full echo, TI 900ms, flip angle 9°). MRI brain imaging acquisition for the UK Biobank included high resolution T1-weighted structural imaging (3D sagittal MPRAGE, 3-Tesla, voxel size 1mm isotropic, 208x256x256 acquisition, TR 2s, TE 2 ms, TI 800ms, flip angle 8°) and T2-weighted imaging (voxel size 1.05x1x1mm, 192x256x256 acquisition).

Supplementary Results

Early and presymptomatic semantic dementia: a case series

Case 1:

A 68-year-old right-handed man presented with a two-and-a-half-year history of evolving apathy, difficulty recognising acquaintances and loss of person-specific knowledge, rigidity of thinking, and increasing difficulties with language. Over this time, he had reduced the frequency of his social interactions. He had lost empathy and emotional attachment to other family members. He found that he struggled to understand subtext in conversations. He was prone to outbursts of temper, including with strangers. He had started gambling on the internet. There was no hyperorality. He increasingly stated that he did not understand the meaning of words and was now unable to complete cryptic crosswords, although had no difficulties with sudoku. He continued to be independent for instrumental daily activities of living, with no decline in mobility or falls.

In the seven and a half years prior to his diagnosis he had been involved in multiple research projects as a healthy volunteer at Cambridge University, including acquisition of repeated structural MRI. Over this time, he had performed strongly in cognitive testing (Fig. 1 and Table 3). There was some evidence of developing behavioural difficulties, with endorsements for difficulties in motivation, cognitive rigidity, sleep, and memory in the CBI-R two and a half years prior to diagnosis.

His cognitive and language testing at point of diagnosis is shown in Supplementary Table 4. He showed deficits in the grading naming test (10/30, 1st-5th percentile), with lower-than-expected verbal IQ given his premorbid functioning. Performance in memory in memory and executive domains was at or above average. His speech was fluent without phonological errors. There was surface dyslexia. He performed poorly on recognition of famous faces.

Given his cognitive profile and imaging features a diagnosis of right semantic dementia was made. He showed the expected progression in the following years given increasingly language involvement and in other cognitive domains. Imaging metrics over time are shown in Supplementary Figure 1.

He died six and half years after diagnosis. Features at postmortem were of frontotemporal lobar degeneration with TDP-43 inclusions, with morphology consistent with subtype C.

Case 2:

A 56-year-old right-handed female was referred to the memory clinic following imaging which showed disproportionate right temporal lobe atrophy. This imaging had been arranged to investigate chronic headaches. In the two years prior to this her informant had noticed that she had difficulties in recognising people's faces. She had become obsessive about her weight and preoccupied with transport timetables. There was hyperorality and a sweet tooth. There was no family history of neurodegenerative disease.

Six-and-a-half-years previously she had been seen by the neurology team with transient sensory disturbance. On review of her imaging, this showed mild right anterior temporal lobe atrophy.

On global cognitive assessment she scored 22/30 on the MMSE and 55/100 on the ACE-R, with deficits in language, fluencies, and memory. There was impaired single word comprehension and object knowledge. She performed poorly on pyramids and palm trees. Sentence repetition and syntactic comprehension were preserved. She could correctly name one out of forty-two famous faces and one out of twelve landmarks. Assessments at her research visit from the FTD clinic are included in Supplementary Table 5.

A diagnosis of right semantic dementia was made. She showed progressive semantic impairment, with evolving impulsive and risky behaviours, and became increasingly dependent. She moved to a residential setting approximately four years post diagnosis.

Case 3:

A 67-year-old left-handed man was referred to the neurology department having been found to have right temporal hypometabolism on an FDG-PET, requested as part of his assessment for a tonsillar cancer. The patient reported no difficulties, except long standing trouble with word finding. An informant described a two-year history of subtle change in decision making, with a tendency to struggle with more complex problems. On occasion he had shown socially inappropriate behaviour, although behavioural changes were not intrusive. They were not aware of any difficulties with facial recognition, language difficulties, or motor problems. There was no relevant family history.

Cognitive testing is outlined in Supplementary Table 5. On global cognitive testing he scored 92/100 on the ACE-R and 30/30 on the MMSE. He performed within average range on tests of verbal and non-verbal memory There was evidence of executive dysfunction, with impaired performance on Raven's coloured progressive matrices B. He showed deficits in famous face matching, landmark name matching, synonym judgement, and social roles judgement. Other tests of semantic general memory were normal. There was no impairment in emotion recognition. There were multiple endorsements on the CBI-R, with a total score of 35. He had no parkinsonism or motor signs.

His imaging features and cognitive profile were in keeping with the early manifestations of the semantic behavioural variant of frontotemporal dementia. On review at 18 months he remained with limited functional deficit, with continued high performance on global cognitive assessment (ACE-R 95/100).

Case 4:

A 67-year-old right-handed man presented to his local memory service with progressive difficulties remembering and finding words over a six-to-twelve-month period, together with struggles with recalling the names of familiar people. There were no reported changes in behaviour. There was minimal impact in his day-to-day functioning. He had a background of hypertension, hypercholesterolaemia, and hypothyroidism.

He had been investigated two years previously for parasthesias in the lower limbs, including an MRI of the brain, which in hindsight showed mild left anterior temporal lobe atrophy. In

addition, he had been investigated for a collapse episode five years prior to this, with no visible atrophy at that point on his imaging.

At his initial assessment he scored 94/100 on the ACE-R, losing points on assessment of fluency and naming, and was given a label of mild cognitive impairment. On return, two years later, this had fallen to 84/100, with primary deficits in the naming language domain. He had 7 endorsements on the CBI-R covering memory and object naming. After a further fourteen months his ACE-R score was 80/100, with impairment on the Sydney Language Battery with difficulties primarily with anomia and word comprehension.

He was given a diagnosis of semantic variant primary progressive aphasia. His assessments around the point of review in the FTD research clinic are included in Supplementary Table 5.

Case 5:

A 67-year-old right-handed man was brought to the attention of the cognitive team following involvement as a healthy volunteer in a multiple sclerosis research study, with MRI Head imaging showing asymmetric, left-side predominant, anterior lobe atrophy. At that point he had an eighteen-month history of difficulty with recalling the correct name or word. There were no significant changes in behaviour. He had no difficulty with recognising faces. He continued to work without concerns raised regarding his performance.

Five years prior to this he had brain imaging as part of investigations for a left parotid tumour, which in retrospect showed mild atrophy in the left temporal pole.

Scores on cognitive testing at diagnosis are shown in Supplementary Table 5. He scored 61/100 on the ACE-R with MMSE of 23/30. There was impaired single word comprehension and had difficulty describing from a verbal definition. He showed non-verbal semantic impairment, scoring 43/52 on pyramids and palm trees. He made multiple errors in recognising famous landmarks. His reading was fluent with multiple regularisation errors. There was no motor impairment, abnormal eye movements, or parkinsonism.

Given his imaging and cognitive features, a diagnosis of semantic variant primary progressive aphasia was made.

Supplementary Figure 1 Longitudinal atrophy profile for Case 1 using the Desikan-Killiany Atlas

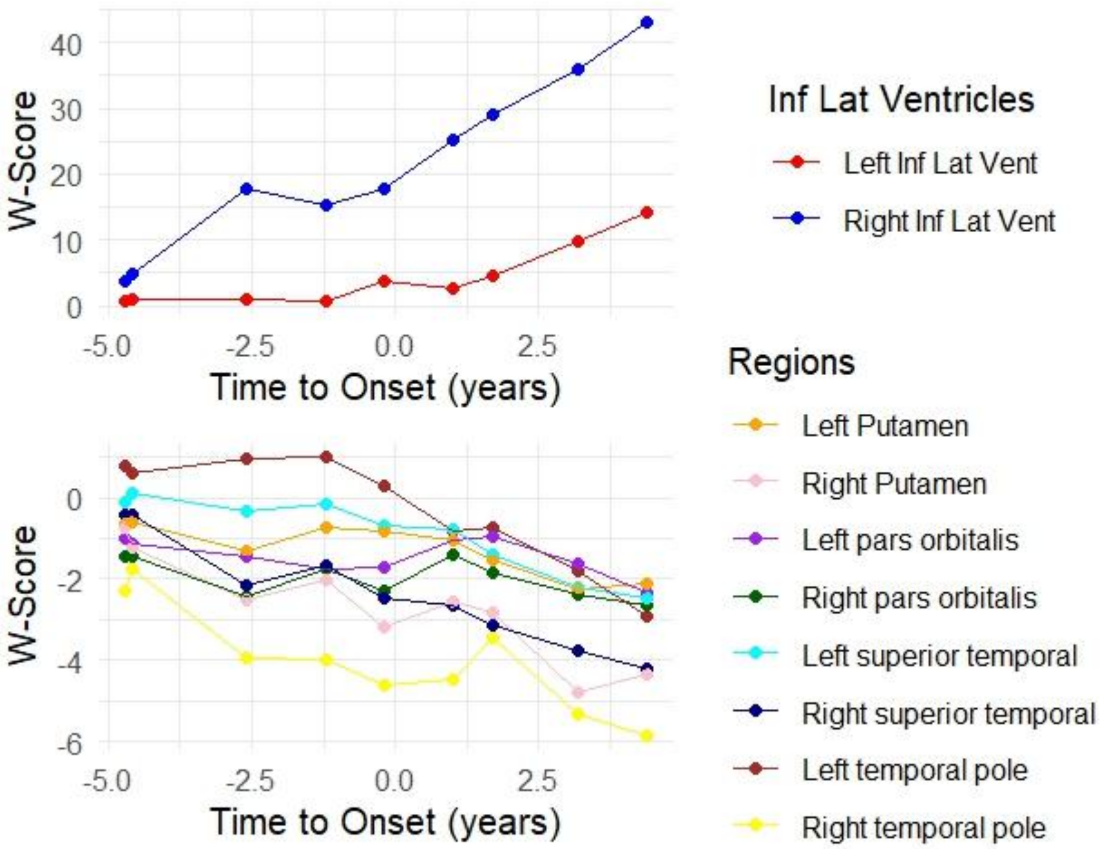

Supplementary Figure 2. T1 weighted MRI imaging for participants with right-lateralised atrophy selected as SD-like from the UK Biobank, reproduced by kind permission of UK Biobank ©

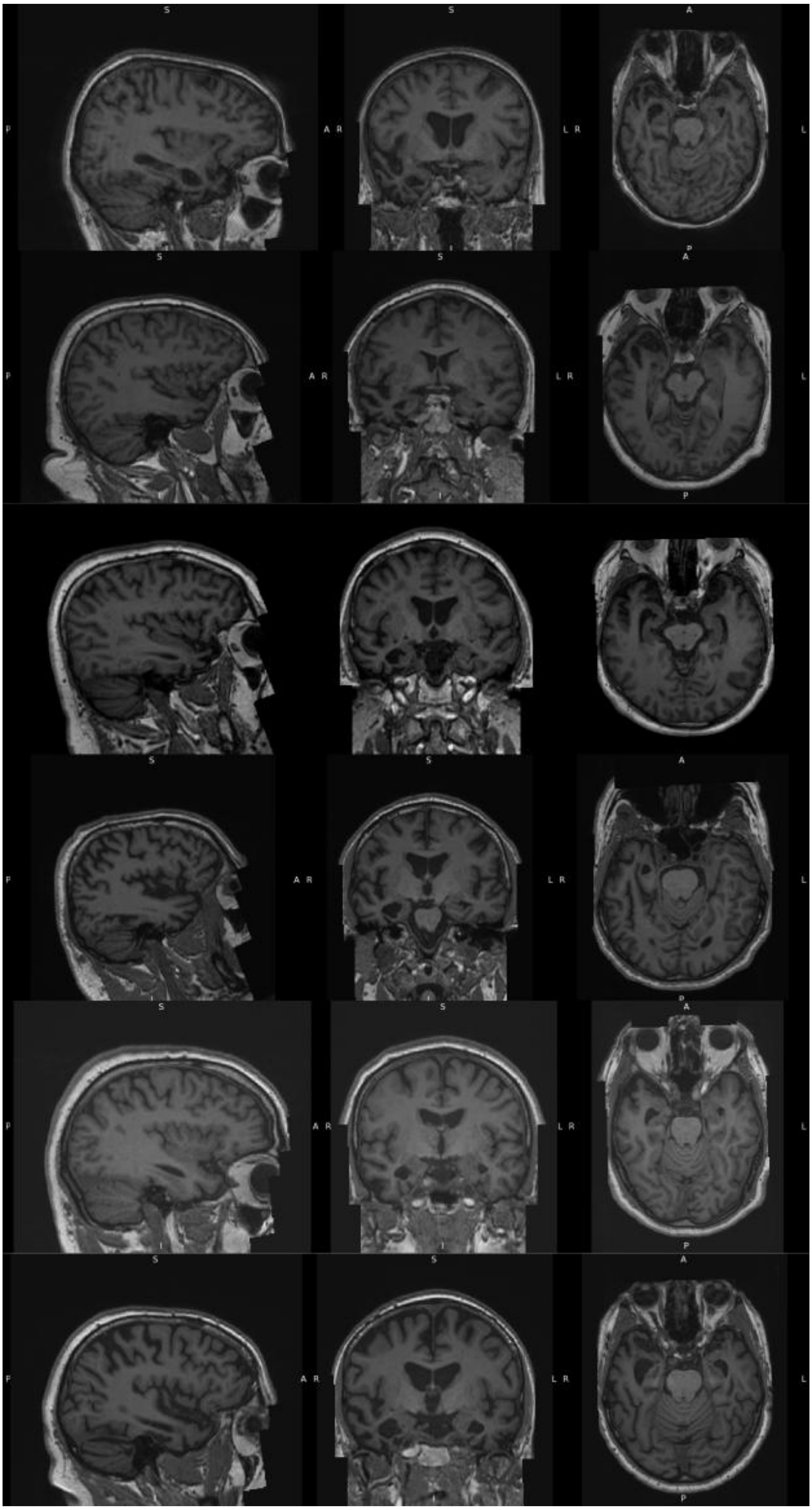

Supplementary Figure 3. T1 weighted MRI imaging for participants with left-lateralised atrophy selected as SD-like from the UK Biobank, reproduced by kind permission of UK Biobank ©

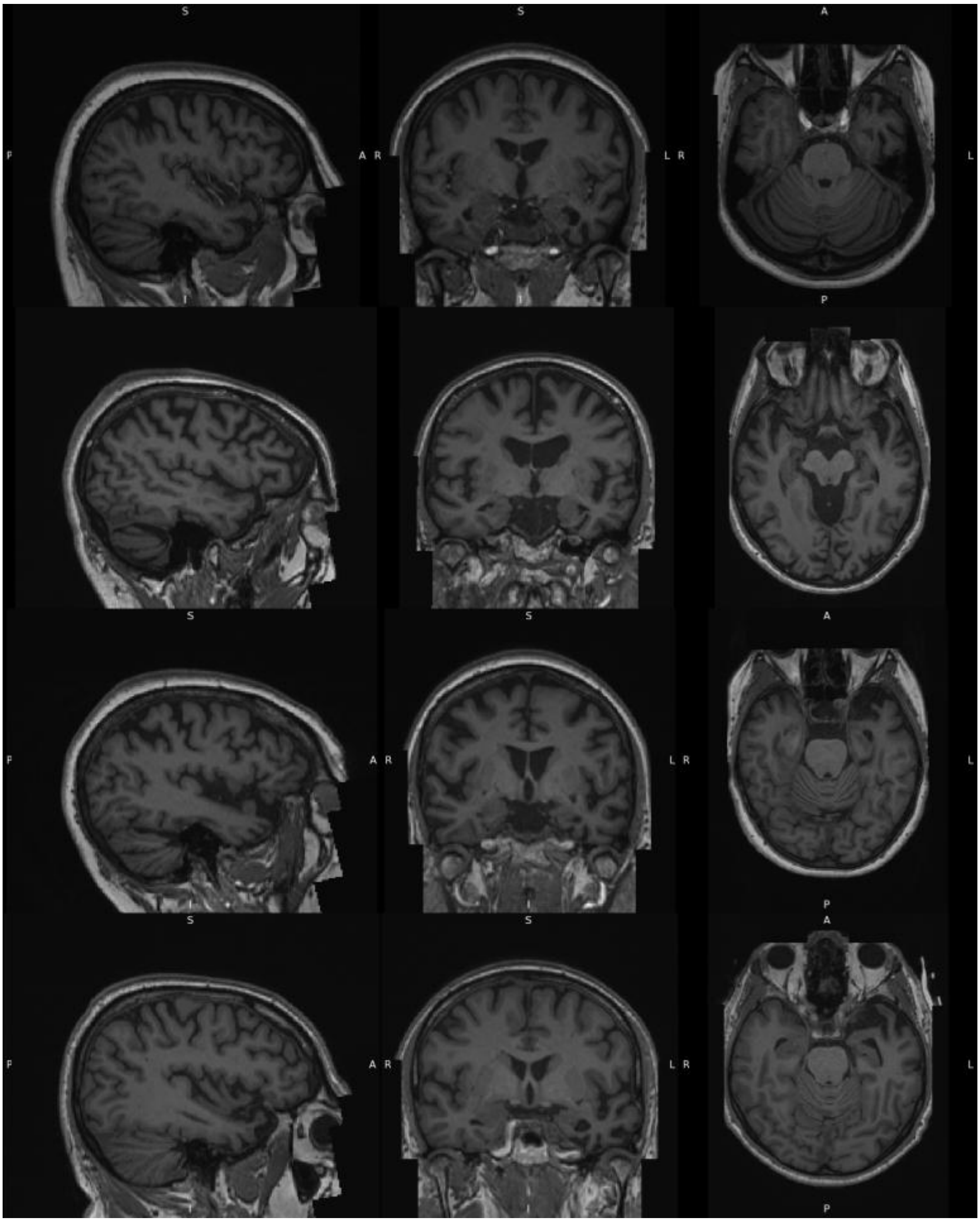

Supplementary Table 1. Demographic and clinical information for non-SD neurodegeneration in the Cambridge cohort

	AD	bvFTD	CBS	DLB	MCI	lvPPA	nfvPPA/ PPAOS	Other PPA	PSP
**Number**	44	63	61	42	31	12	38	17	190
**Sex (female/male)**	15/29	22/41	34/27	8/34	12/19	8/4	18/20	11/6	84/106
**Age at baseline imaging, mean (sd)**	72.9 (7.9)	63.9 (7.7)	69.5 (7.5)	76.1 (6.4)	75.4 (8.0)	70.5 (7.6)	74.4 (7.3)	74.6 (7.4)	72.2 (7.1)
**Disease duration at baseline imaging, mean (sd)**	5.1 (2.7)	4.7 (2.4)	3.7 (2.3)	4.9 (3.8)	3.2 (2.2)	4.1 (1.3)	3.7 (1.9)	3.9 (3.0)	4.3 (2.7)
**ACE-R/ACE-III /100, mean (sd, n)**	68.7 (15, 44)	62.9 (23, 63)	69.2 (22, 60)	67.8 (17, 40)	82 (6.3, 30)	43.8 (24, 12)	70.3 (18, 38)	62.0 (18, 17)	77.9 (14, 188)
**CBI-R, mean (sd, n)**	52.6 (26, 31)	82.8 (30, 55)	44.8 (31, 54)	54.0 (27, 38)	25.6 (17, 24)	37.3 (24, 11)	31.3 (29,33)	45.6 (25, 13)	48.6 (32, 151)

Supplementary Table 2. MRI models and acquisition parameters for the Cambridge cohort

Device	Number	Group	Slice thickness (mm)	Echo time (ms)	Repetition time (s)	Inversion time (s)	Flip angle (degree)
**WBIC Prisma**	217	SD n=8<br>Non-SD n=209	1.1	2.93	2	0.85	8
**Skyra**	44	SD n=7<br>Non-SD n=37	1-1.1	2.86 – 2.98	2-2.3	0.9	8-9
**Tim Trio**	239	SD n=25<br>Non-SD n=214	1-1.25	2.86 – 2.98	2.3	0.8	9-10
**Verio**	71	Non-SD n=71	1	2.98	2-2.3	0.9	9
**Sigma**	55	Non-SD n=65	1	3.4	0.009	0.45	12
**CBU Prisma**	6	SD n=6	1	2.91	2.3	0.9	9
**CBU Tim Trio**	1	SD n=1	1	2.98	2.25	0.9	9

SD Semantic dementia, Non-SD other neurodegenerative conditions or healthy controls, WBIC Wolfson Brain Imaging Centre, CBU MRC Cognition and Brain Sciences Unit

Supplementary Table 3. Model family and prior for assessment of cognitive function and general overall health in the UK Biobank

Test/Rating	Model family	Prior
General Health Rating	Cumulative	Normal(0, 1)
Alphanumeric trail making	Lognormal	Normal(0, log(1+ (SD/mean)))
Digit Symbol	Gaussian	Normal(0, 1)
Tower Rearanging	Gaussian	Normal(0, 1)
Matrix Pattern Completion	Gaussian	Normal(0, 1)
Fluid Intelligence	Gaussian	Normal(0, 1)
Picture Naming	Skew Normal	Normal(0, 1)
Paired Associate Learning	Truncated Gaussian	Normal(0, SD)
Digit span	Gaussian	Normal(0, 1)
Pairs matching (round 1 +2)	Negative Binomial	Normal(0, log(1+ (SD/mean)))
Reaction time	Shifted lognormal	Normal(0, log(1+ (SD/mean)))
Prospective Memory	Cumulative	Normal(0, 1)

SD Standard deviation

Supplementary Table 4 Cognitive profile at diagnosis for case 1

Test	Score (total/scaled/z-score)	Percentile
ACE-R	96	
NART	Predicted FSIQ 127	
**WAIS**		
Verbal IQ	103	58
Performance IQ	124	95
Full scale IQ	112	79
Verbal comprehension index	108	75
Perceptual orientation index	117	78
Working memory index	103	47
Processing speech index	120	93
**WMS -stories**		
Immediate recall	11	63
Delayed recall	12	75
**WMS – list learning**		
Immediate total recall	12	75
Learning slope	9	37
Long delay recall	14	91
Recognition	11	63
Percentage retention	15	95
**Rey figure**		
Copy	0.8	79
Delayed recall	2.2	98
**Graded naming test**	10	1-5
**Doors and People**	9	25-50
**Trails**		
Trail A	36	30-40
Trail B	68	50

ACE-R Addenbrooke's Cognitive Examination-Revised; NART National Adult Reading Test; WAIS Weschler Adult Intelligence Scale

Supplementary Table 5 Cognitive profile at first FTLD research visit for cases 2-5

Test	Case 2	Case 3	Case 4	Case 5
MMSE (/30)	29	30	**24**	29
ACE-R (/100)	**66**	92	**65**	**75**
ACE-R Attention (/18)	17	18	15	18
ACE-R Memory (/26)	14	21	16	20
ACE-R Fluency (/14)	8	11	5	9
ACE-R Language (/26)	12	26	13	12
ACE-R Visuospatial (/16)	15	16	16	16
Brixton	8	-	6	6
Raven's B (/12)	12	**7**	12	**6**
Trails A (sec)	-	46	24	50
Trails B (sec)	-	121	56	105
Cambridge Naming (/32)	**20**	32	**20**	**19**
Boston Naming (/30)	**8**	30	**11**	**9**
Camel and Cactus (/32)	**17**	31	**16**	**21**
Synonym Judgement (/48)	**42**	**45**	**41**	**41**
Word-picture matching (/36)	36	36	**33**	**35**
Social roles (/35)	**30**	**32**	**27**	**31**
Face-name matching (/44)	**12**	39	**14**	40
Landmark-name matching (/42)	**12**	**31**	**22**	**28**
Familiar face matching (/22)	**19**	**19**	**19**	20
Unfamiliar face matching (/18)	19	18	20	19
Social norms questionnaire (/22)	**16**	18	**15**	20
CBI-R	**111**	**35**	12	6

Neurocognitive testing and behavioural rating scales for cases 2-5 at their first FTLD research visit. Numbers in bold represent differences from standardised cut offs as set for cognitive impairment for the MMSE, ACE-R, and Trail Making Task. For the CBI-R control data is taken from. For other tasks (except the ACE-R subscales) differences are using the modified Crawford t-test against a control sample of 19 individuals. Details of the control sample and testing are in Rouse et al.[1] ACE-R Addenbrooke's Cognitive Examination-Revised; MMSE Mini Mental State Examination; CBI-R Cambridge Behavioural Inventory-Revised

Supplementary Table 6 Performance metrics across datasets at the primary threshold

Dataset	Accuracy	Recall	Specificity	Precision	NPV	F1 score
Cambridge	0.98	0.92	0.98	0.83	0.99	0.87
NIFD + ADNI 3	0.99	0.88	0.997	0.95	0.99	0.91

Metrics for the Cambridge dataset are derived through repeated cross-validation. NIFD Neuroimaging in frontotemporal dementia; ADNI Alzheimer's Dementia Neuroimaging Initiative, NPV negative predictive value.

Supplementary Table 7 Highest ranking features by permutation importance for the NIFD + ADNI 3 dataset

Feature	Importance
Minimum w-score differences from temporal pole to non-temporal lobe cortical regions	0.11
Amygdala minimum w-score	0.066
Temporal pole minimum w-score	0.047
Enterohinal minimum w-score	0.043
Minimum w-score differences from middle temporal gyrus to non-temporal lobe cortical regions	0.034
Superior temporal w-score hemispheric difference	0.017
Middle temporal w-score hemispheric difference	0.014
Parahippocampal gyrus minimum w-score	0.012
Inferior lateral ventricles w-score hemispheric difference	0.010
Insula minimum w-score	0.0085

The ten highest ranking features by permutation importance for F1 score for the combined NIFD + ADNI 3 test set. ADNI: Alzheimer's Disease Neuroimaging Initative, NIFD: Frontotemporal lobar degeneration neuroimaging initiative

Supplementary Table 8 Performance metrics across datasets at threshold 0.38

Dataset	Accuracy	Recall	Specificity	Precision	NPV	F1 score
Cambridge	0.98	0.95	0.98	0.81	0.996	0.87
NIFD + ADNI 3	0.99	0.90	0.99	0.90	0.99	0.90

Supplementary Table 9 Performance metrics across datasets with features of cross-hemisphere differences removed

Dataset	Accuracy	Recall	Specificity	Precision	NPV	F1 score
Cambridge	0.97	0.89	0.98	0.76	0.99	0.82
NIFD + ADNI 3	0.98	0.88	0.98	0.78	0.99	0.83

Performance metrics at threshold 0.44, number of estimators n=100, maximum depth n=4

Supplementary Table 10 Cognitive testing for the SD group at the primary threshold

	Bayesian model			Frequentist model		
**Cognitive/health assessment**	**Estimated Beta**	**CI**	**Bayes Factor**	**Estimate**	***t/z* value**	***P* value**
Health rating	0.53	-0.53 – 1.6	1.2	0.77	1.2	0.23
Fluid Intelligence	-0.6	-1.19 - 0	0.51	-0.66	-2.1	0.035
**Picture naming**	**-0.71**	**-1.32 - -0.13**	**0.19**	**-0.81**	**-2.5**	**0.012**
Matrix completion	-0.22	-0.85 – 0.39	2.5	-0.25	-0.74	0.46
Trails B	0.15	-0.06 – 0.37	1.58	0.16	1.4	0.17
Digit symbol	-0.49	-1.09 – 0.08	0.89	-0.54	-1.7	0.089
Tower arranging	-0.12	-0.85 – 0.57	2.5	-0.14	-0.26	0.72
Reaction time	0.01	-0.12 – 0.13	2.57	-0.03	-0.001	0.99
**Prospective memory**	**1.85**	**0.79 – 2.85**	**0.01**	**2.6**	**4.3**	**0.0002**
Pairs matching	0.38	-0.01 – 0.79	0.59	0.41	2.1	0.038
Paired associate learning	-0.12	-3.7 – 3.59	1.45	-0.13	-0.15	0.88
Digit span	0.45	-0.3 – 1.15	1.3	0.52	1.3	0.19

Supplementary Table 11 Cognitive testing for the SD group for a model defined without features of cross-hemispheric differences in volume

	Bayesian model			Frequentist model		
**Cognitive/health assessment**	**Estimated Beta**	**CI**	**Bayes Factor**	**Estimate**	***t*/*z* value**	***P* value**
**Health rating**	**1.0**	**0.14 – 1.87**	**0.18**	**1.26**	**2.6**	**0.008**
**Fluid Intelligence**	**-0.59**	**-1.0 - -0.14**	**0.17**	**-0.62**	**-2.5**	**0.012**
**Picture naming**	**-0.71**	**-1.2 - -0.26**	**0.04**	**-0.79**	**-3.2**	**0.0015**
Matrix completion	-0.44	-0.9 – 0.03	0.79	-0.47	-1.9	0.061
Trails B	0.17	0 – 0.34	0.59	0.18	2.0	0.041
Digit symbol	-0.44	-0.88 – 0.02	0.81	-0.46	-1.9	0.053
Tower arranging	-0.06	-0.63 – 0.51	3.3	-0.06	-0.20	0.84
Reaction time	0.08	-0.01 – 0.18	0.88	0.07	1.9	0.058
**Prospective memory**	**1.78**	**0.96 – 2.57**	**0.000001**	**2.15**	**4.7**	**0.000002**
Pairs matching	0.29	0 – 0.68	0.68	0.20	1.4	0.17
Paired associate learning	-2.0	-4.8 – 0.9	0.71	-1.2	-1.8	0.073
Digit span	-0.35	-0.92 – 0.21	1.74	-0.38	-1.3	0.21

Supplementary Table 12 Cognitive testing for the SD group at the lower threshold (0.38)

	Bayesian model			Frequentist model		
**Cognitive/health assessment**	**Estimated Beta**	**CI**	**Bayes Factor**	**Estimate**	***t/z* value**	***P* value**
Health rating	0.84	-0.03 – 1.71	0.37	1.1	2.2	0.031
**Fluid Intelligence**	**-0.74**	**-1.24 - -0.23**	**0.05**	**-0.79**	**-3.0**	**0.0027**
**Picture naming**	**-0.56**	**-1.04 - -0.09**	**0.27**	**-0.64**	**-2.5**	**0.014**
Matrix completion	-0.47	-0.96 – 0.02	0.78	-0.51	-1.9	0.051
Trails B	0.17	0 – 0.34	0.66	0.18	1.97	0.049
**Digit symbol**	**-0.51**	**-0.98 - -0.03**	**0.32**	**-0.54**	**-2.2**	**0.029**
Tower arranging	-0.16	-0.72 – 0.4	2.96	-0.17	-0.56	0.58
Reaction time	0.09	-0.01- 0.19	0.83	0.08	1.9	0.06
**Prospective memory**	**2.0**	**1.2 – 2.9**	**0.0008**	**2.5**	**5.2**	**0.0000003**
Pairs matching	0.32	0.01 – 0.65	0.53	0.32	2.1	0.040
Paired associate learning	-1.71	-4.7 – 1.39	0.96	-0.98	-1.4	0.16
Digit span	-0.08	-0.65 – 0.49	3.1	-0.08	-0.26	0.80

1. Rouse MA, Halai AD, Ramanan S, et al. Social-semantic knowledge in frontotemporal dementia and after anterior temporal lobe resection. *Brain Communications*. 2024;6(6):fcae378. doi:10.1093/braincomms/fcae378
